# Synthesis and crystal structure of bis­(μ-2-methyl­benzene­thiol­ato-κ^2^ *S*:*S*)bis­[meth­yl(2-methyl­benzene­thiol­ato-κ*S*)indium(III)]

**DOI:** 10.1107/S2056989017003498

**Published:** 2017-03-10

**Authors:** Glen G. Briand, Andreas Decken, Courtney M. Dickie, Gregory MacNeil

**Affiliations:** a Department of Chemistry and Biochemistry, Mount Allison University, 63C York Street, Sackville, NB, E4L 1G8, Canada; b Department of Chemistry, University of New Brunswick, Fredericton, NB, E3B 5A3, Canada

**Keywords:** crystal structure, indium, thiol­ate, dinuclear, coordination polymer

## Abstract

The dinuclear title compound, [In_2_(CH_3_)_2_(C_7_H_7_S)_4_] or [Me(2-MeC_6_H_4_S)In-μ-(2-MeC_6_H_4_S)_2_InMe(2-MeC_6_H_4_S)], was prepared from the 1:2 reaction of Me_3_In and 2-MeC_6_H_4_SH in toluene. Its crystal structure exhibits a four-membered In_2_S_2_ ring core *via* bridging (2-MeC_6_H_4_S) groups. The dimeric units are further associated into a one-dimensional polymeric structure extending parallel to the *a* axis *via* inter­molecular In⋯S contacts. The In atoms are then in distorted trigonal–bipyramidal CS_4_ bonding environments.

## Abstract 2

The dinuclear compound, [Me(2-MeC_6_H_4_S)In-μ-(2-MeC_6_H_4_S)_2_InMe(2-MeC_6_H_4_S)], was prepared from the 1:2 reaction of Me_3_In and 2-MeC_6_H_4_SH in toluene. Its crystal structure exhibits a four-membered In_2_S_2_ ring core *via* bridging (2-MeC_6_H_4_S) groups. The dimeric units are further associated into a one-dimensional polymeric structure *via* inter­molecular In⋯S contacts.

## Chemical context   

Methyl­indium di­thiol­ates [MeIn(S_2_ *R*)] have been shown to be useful compounds for the ring-opening polymerization (ROP) of cyclic esters to produce biodegradable polymers (Allan *et al.*, 2013 ▸; Briand *et al.*, 2016 ▸). These compounds are prepared from the stoichiometric reaction of InMe_3_ with polydentate amino/oxo-di­thiols. However, the 1:2 reaction of triorganyl­indium (*R*_3_In) with simple mono­thiols (*R*′SH) often results in isolation of the diorganylindium thiol­ate *R*_2_In(S*R*′) (Hoffmann, 1988 ▸; Nomura *et al.*, 1989 ▸). The favourable formation of the organylindium di­thiol­ate *R*In(S*R*′)_2_ was reported to be determined by the steric bulk of the thiol­ate ligand and the *R*-In group, and the acidity of the thiol reactant. The 1:2 reaction of *n*Bu_3_In or *i*Bu_3_In and PhSH afforded the di­thiol­ate *R*In(SPh)_2_ (*R* = *n*Bu, *i*Bu) as solids, although the compounds were poorly soluble in organic solvents, precluding crystallization. All compounds in these studies were primarily characterized by NMR. The only structurally characterized example of such a compound is [(Me_3_Si)_3_C](PhS)In-μ-(PhS)_2_In[C(Me_3_Si)_3_](SPh), which is prepared from the redox reaction of the indium(I) compound [(Me_3_Si)_3_CIn]_4_ and the di­sulfide (SPh)_2_ (Peppe *et al.*, 2009 ▸). The 1:2 reaction of Me_3_In and 2-MeC_6_H_4_SH in toluene affords [Me(2-MeC_6_H_4_S)In-μ-(2-MeC_6_H_4_S)_2_InMe(2-MeC_6_H_4_S)], (I), in high yield. The modest steric bulk afforded by the 2-MeC_6_H_4_ group moderates inter­molecular bonding and increases solubility in organic solvents without preventing formation of the *R*In(S*R*′)_2_ species. The observation of only one signal for the *Me*In and 2-*Me*C_6_H_4_S groups in the ^1^H NMR study suggests that the compound dissociates into MeIn(2-*Me*C_6_H_4_S)_2_ monomers in tetrahydrofuran solution.
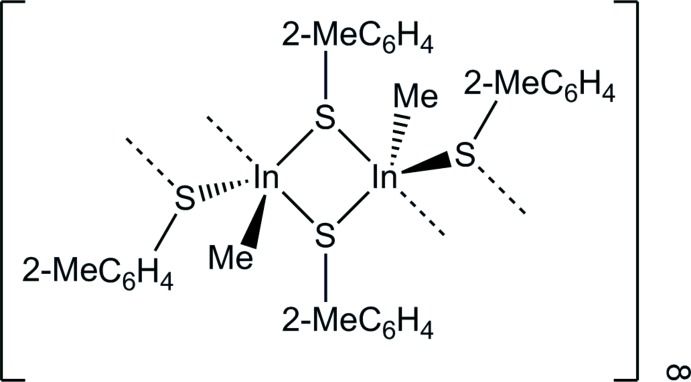


## Structural commentary   

**Figure 1:**
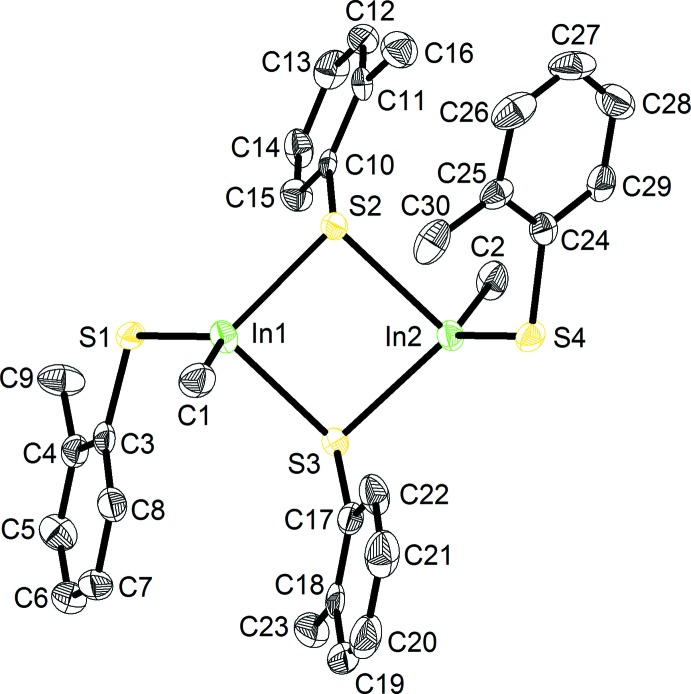
The asymmetric unit of (I), with displacement ellipsoids drawn at the 50% probability level. H atoms have been omitted for clarity.

The asymmetric unit comprises the dinuclear compound, [Me(2-MeC_6_H_4_S)In-μ-(2-MeC_6_H_4_S)_2_InMe(2-MeC_6_H_4_S)], (I) (Fig. 1 ▸). The two unique indium atoms are each bonded to a methyl carbon atom, and one terminal and one bridging (2-MeC_6_H_4_S) group, generating a nearly square-planar four-membered In_2_S_2_ ring core [S2—In1—S3 = 88.28 (6), In1—S2—In2 = 91.86 (6), S2—In2—S3 = 87.02 (6), In1—S3—In2 = 92.58 (7)°]. The In atoms are in distorted trigonal–pyramidal CS_3_ bonding environments [C1—In1—S1 = 127.3 (2), C1—In1—S2 = 113.1 (3), S1—In1—S2 = 114.66 (7), C1—In1—S3 = 105.7 (2), S1—In1—S3 = 96.94 (6), S2—In1—S3 = 88.28 (6), C2—In2—S3 = 118.2 (3), C2—In2—S4 = 124.1 (3), S3—In2—S4 = 115.00 (7), C2—In2—S2 = 102.4 (2), S2—In2—S3 = 87.02 (6), S2—In2—S4 = 95.87 (6)°]. Bond lengths and angles are similar at each indium atom.

## Supra­molecular features   

**Figure 2:**
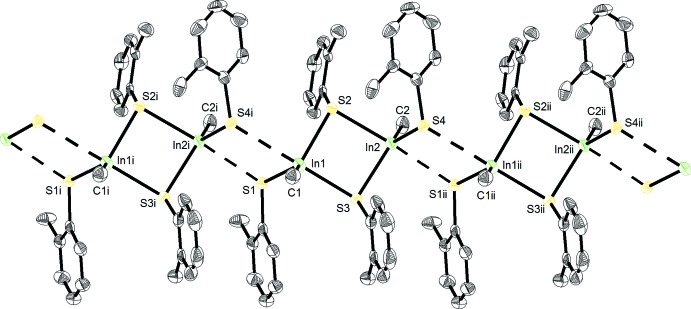
Part of the crystal structure of (I), with displacement ellipsoids drawn at the 50% probability level. H atoms have been omitted for clarity. [Symmetry codes: (i) −1 + *x*, *y*, *z*; (ii) 1 + *x*, *y*, *z*.]

The dimeric structures are further associated into one-dimensional polymers extending parallel to the *a* axis *via* inter­molecular In⋯S contacts [In1⋯S4(*x* − 1, *y*, *z*) = 3.091 (2), In2⋯S1(*x* + 1, *y*, *z*) = 2.920 (2) Å] (sum of metallic/van der Waals radii = 3.52 Å; Bondi, 1964 ▸) (Fig. 2 ▸). Such contacts are common for indium and other heavy main group metal chalcogenolates due to their large metal radii and potential for high coordination numbers (Briand *et al.*, 2010 ▸, 2011 ▸, 2012 ▸; Appleton *et al.*, 2011 ▸). This leads to the formation of insoluble materials for *i*BuIn(SPh)_2_ (Nomura *et al.*, 1989 ▸). The steric bulk provided by the Me group of the (2-MeC_6_H_4_S) ligand is sufficient to moderate inter­molecular contacts and afford solubility in organic solvents (*e.g*. toluene and tetra­hydro­furan).

## Database survey   

The dinuclear structure of (I) is similar to that of [Me(MeO_2_CCH_2_CH_2_S)In-μ-(MeO_2_CCH_2_CH_2_S)_2_InMe(MeO_2_CCH_2_CH_2_S)] (Allan *et al.*, 2013 ▸). However, the ester carbonyl oxygen atoms of the terminal MeO_2_CCH_2_CH_2_S groups occupy the coordination site *trans* to the axial bridging thiol­ate sulfur atom. This precludes inter­molecular In⋯S bonding and yields discrete dimeric units. The structure of (I) is also similar to that of the structure of dimeric [(Me_3_Si)_3_C](PhS)In-μ-(PhS)_2_In[C(Me_3_Si)_3_](SPh) (Peppe *et al.*, 2009 ▸). However, the steric bulk of the (Me_3_Si)_3_C precludes further inter­molecular In⋯S bonding and the indium atoms are restricted to a four-coordinate distorted tetra­hedral bonding environment. Other reported methyl­indium di­thiol­ates employ polydentate di­thiol­ate ligands, some of which possess dimeric and trimeric structures (Briand *et al.*, 2016 ▸).

## Synthesis and crystallization   

2-Methyl­benzene­thiol (0.300 g, 2.42 mmol) in toluene (2 ml) was added dropwise to a stirred solution of InMe_3_ (0.193 g, 1.21 mmol) in toluene (5 ml). The solution was stirred for 18 h and concentrated *in vacuo* to 4 ml. After sitting at 296 K for 1 d, the solution was filtered to yield colourless, needle-like crystals of (I). Yield: 0.317 g (0.421 mmol, 70%). Analysis calculated for C_30_H_34_S_4_In_2_: C, 47.88; H, 4.55; N, 0.00. Found: C, 46.88; H, 4.55; N, <0.3. M.p 421–422 K.

FT—IR (cm^−1^): 672 *s*, 705 *s*, 741 *s*, 800 *w*, 846 *w*, 861 *w*, 939 *w*, 978 *w*, 1041 *m*, 1055 *m*, 1280 *w*, 1378 *w*, 1451 *m*, 1464 *m*, 1585 *w*, 2913 *w*, 3056 *w*. FT–Raman (cm^−1^): 121 *vs*, 158 *s*, 244 *w*, 322 *m*, 443 *w*, 508 *s*, 552 *w*, 675 *w*, 800 *m*, 1043 *s*, 1128 *w*, 1148 *w*, 1204 *m*, 1465 *w*, 1565 *w*, 1586 *m*, 2916 *w*, 3047 *m*. ^1^H NMR (200 MHz, thf-*d*_8_, p.p.m.): δ = 0.23 [*s*, 3H, *Me*In], 2.60 [*s*, 6H, (S-2-*Me*C_6_H_4_)], 7.06–7.11 [*m*, 4H, (S-2-MeC_6_ *H*_4_)] 7.23–7.28 [*m*, 2H, (S-2-MeC_6_ *H*_4_)], 7.62–7.66 [*m*, 2H, (S-2-MeC_6_ *H*_4_)]. ^13^C{^1^H} NMR (101 MHz, thf-*d*_8_, p.p.m.): δ = −5.1 (*Me*In), 21.7 (S-2-*Me*C_6_H_4_), 124.1, 125.2, 129.4, 134.6, 138.4, 139.7 (S-2-Me*C*_6_H_4_)].

## Refinement   

Crystal data, data collection and structure refinement details are summarized in Table 1 ▸. H atoms were included in calculated positions and refined using a riding model.

## Supplementary Material

Crystal structure: contains datablock(s) I. DOI: 10.1107/S2056989017003498/lh5837sup1.cif

Structure factors: contains datablock(s) I. DOI: 10.1107/S2056989017003498/lh5837Isup2.hkl

CCDC reference: 1535922

Additional supporting information:  crystallographic information; 3D view; checkCIF report

## Figures and Tables

**Table 1:** Experimental details

Crystal data	
Chemical formula	[In_2_(CH_3_)_2_(C_7_H_7_S)_4_]
*M*_r_	752.45
Crystal system, space group	Monoclinic, *P*2_1_
Temperature (K)	173
*a*, *b*, *c* (Å)	7.4441 (15), 14.625 (3), 14.074 (3)
β (°)	99.693 (3)
*V* (Å^3^)	1510.4 (5)
*Z*	2
Radiation type	Mo *K*α
μ (mm^−1^)	1.82
Crystal size (mm)	0.45 × 0.08 × 0.03

Data collection	
Diffractometer	Bruker *SMART1000*/P4
Absorption correction	Multi-scan (*SADABS*; Sheldrick, 2008*a* ▸)
*T*_min_, *T*_max_	0.495, 0.956
No. of measured, independent and observed [*I* > 2σ(*I*)] reflections	10442, 5591, 4742
*R*_int_	0.041
(sin θ/λ)_max_ (Å^−1^)	0.650

Refinement	
*R*[*F* ^2^ > 2σ(*F* ^2^)], *wR*(*F* ^2^), *S*	0.034, 0.074, 1.04
No. of reflections	5591
No. of parameters	332
No. of restraints	1
H-atom treatment	H-atom parameters constrained
Δρ_max_, Δρ_min_ (e Å^−3^)	0.49, −1.01
Absolute structure	Flack (1983 ▸), 2079 Friedel pairs
Absolute structure parameter	0.41 (3)

Computer programs: *SMART* (Bruker, 1999 ▸), *SAINT* (Bruker, 2006 ▸), *SHELXS97* and *SHELXTL* (Sheldrick, 2008*b* ▸), *SHELXL2013* (Sheldrick, 2015 ▸) and *DIAMOND* (Brandenburg, 2012 ▸).

## supplementary crystallographic information

### Crystal data

**Table:**

[In_2_(CH_3_)_2_(C_7_H_7_S)_4_]	*F*(000) = 752
*M**_r_* = 752.45	*D*_x_ = 1.655 Mg m^−^^3^
Monoclinic, *P*2_1_	Mo *K*α radiation, λ = 0.71073 Å
*a* = 7.4441 (15) Å	Cell parameters from 5877 reflections
*b* = 14.625 (3) Å	θ = 2.8–27.8°
*c* = 14.074 (3) Å	µ = 1.82 mm^−^^1^
β = 99.693 (3)°	*T* = 173 K
*V* = 1510.4 (5) Å^3^	Rod, colourless
*Z* = 2	0.45 × 0.08 × 0.03 mm

### Data collection

**Table:**

Bruker SMART1000/P4 diffractometer	5591 independent reflections
Radiation source: fine-focus sealed tube, K760	4742 reflections with *I* > 2σ(*I*)
Graphite monochromator	*R*_int_ = 0.041
φ and ω scans	θ_max_ = 27.5°, θ_min_ = 1.5°
Absorption correction: multi-scan (SADABS; Sheldrick, 2008a)	*h* = −9→9
*T*_min_ = 0.495, *T*_max_ = 0.956	*k* = −18→19
10442 measured reflections	*l* = −18→17

### Refinement

**Table:**

Refinement on *F*^2^	Secondary atom site location: difference Fourier map
Least-squares matrix: full	Hydrogen site location: inferred from neighbouring sites
*R*[*F*^2^ > 2σ(*F*^2^)] = 0.034	H-atom parameters constrained
*wR*(*F*^2^) = 0.074	*w* = 1/[σ^2^(*F*_o_^2^) + (0.0276*P*)^2^] where *P* = (*F*_o_^2^ + 2*F*_c_^2^)/3
*S* = 1.04	(Δ/σ)_max_ = 0.001
5591 reflections	Δρ_max_ = 0.49 e Å^−^^3^
332 parameters	Δρ_min_ = −1.01 e Å^−^^3^
1 restraint	Absolute structure: Flack (1983), 2079 Friedel pairs
Primary atom site location: structure-invariant direct methods	Absolute structure parameter: 0.41 (3)

### Special details

**Table:**

Experimental. Crystal decay was monitored by repeating the initial 50 frames at the end of the data collection and analyzing duplicate reflections.
Geometry. All esds (except the esd in the dihedral angle between two l.s. planes) are estimated using the full covariance matrix. The cell esds are taken into account individually in the estimation of esds in distances, angles and torsion angles; correlations between esds in cell parameters are only used when they are defined by crystal symmetry. An approximate (isotropic) treatment of cell esds is used for estimating esds involving l.s. planes.
Refinement. Refined as a 2-component inversion twin

### Fractional atomic coordinates and isotropic or equivalent isotropic displacement parameters (Å^2^)

**Table:**

	*x*	*y*	*z*	*U*_iso_*/*U*_eq_	
In1	0.60776 (7)	0.80291 (3)	0.26699 (4)	0.02518 (14)	
In2	1.05764 (7)	0.69727 (3)	0.23677 (4)	0.02406 (14)	
S1	0.4196 (2)	0.70588 (16)	0.35474 (14)	0.0268 (4)	
S2	0.7227 (3)	0.72420 (11)	0.12869 (14)	0.0218 (4)	
S3	0.9290 (3)	0.76607 (13)	0.37732 (15)	0.0232 (4)	
S4	1.2193 (2)	0.80568 (17)	0.14747 (13)	0.0268 (4)	
C1	0.5973 (11)	0.9472 (6)	0.2527 (7)	0.036 (2)	
H1A	0.6958	0.9746	0.2988	0.054*	
H1B	0.4796	0.9694	0.2656	0.054*	
H1C	0.6115	0.9641	0.1870	0.054*	
C2	1.0874 (11)	0.5515 (5)	0.2317 (7)	0.034 (2)	
H2A	1.2104	0.5364	0.2206	0.051*	
H2B	0.9975	0.5265	0.1792	0.051*	
H2C	1.0678	0.5249	0.2931	0.051*	
C3	0.4643 (10)	0.7328 (5)	0.4795 (6)	0.0235 (17)	
C4	0.4170 (10)	0.6667 (5)	0.5433 (6)	0.0269 (18)	
C5	0.4608 (11)	0.6852 (6)	0.6420 (6)	0.0346 (19)	
H5	0.4308	0.6413	0.6865	0.042*	
C6	0.5449 (11)	0.7639 (6)	0.6763 (6)	0.035 (2)	
H6	0.5751	0.7736	0.7439	0.042*	
C7	0.5863 (11)	0.8296 (5)	0.6137 (6)	0.034 (2)	
H7	0.6436	0.8850	0.6376	0.040*	
C8	0.5435 (10)	0.8145 (6)	0.5147 (5)	0.0297 (18)	
H8	0.5687	0.8604	0.4711	0.036*	
C9	0.3221 (13)	0.5794 (6)	0.5076 (6)	0.041 (2)	
H9A	0.2848	0.5467	0.5618	0.061*	
H9B	0.4054	0.5410	0.4780	0.061*	
H9C	0.2143	0.5937	0.4597	0.061*	
C10	0.6499 (9)	0.6072 (5)	0.1171 (5)	0.0196 (16)	
C11	0.6429 (10)	0.5650 (5)	0.0265 (6)	0.0254 (17)	
C12	0.6019 (10)	0.4720 (5)	0.0215 (6)	0.0307 (19)	
H12	0.5984	0.4414	−0.0383	0.037*	
C13	0.5667 (11)	0.4229 (5)	0.0989 (6)	0.035 (2)	
H13	0.5361	0.3599	0.0918	0.042*	
C14	0.5755 (11)	0.4651 (5)	0.1872 (6)	0.0305 (19)	
H14	0.5540	0.4311	0.2417	0.037*	
C15	0.6163 (11)	0.5583 (5)	0.1957 (6)	0.0285 (18)	
H15	0.6208	0.5881	0.2560	0.034*	
C16	0.6849 (12)	0.6168 (6)	−0.0591 (6)	0.037 (2)	
H16A	0.8122	0.6372	−0.0464	0.056*	
H16B	0.6045	0.6701	−0.0708	0.056*	
H16C	0.6656	0.5770	−0.1159	0.056*	
C17	1.0334 (10)	0.8758 (5)	0.4071 (6)	0.0281 (18)	
C18	1.0799 (11)	0.8983 (6)	0.5038 (7)	0.038 (2)	
C19	1.1513 (12)	0.9872 (7)	0.5242 (8)	0.050 (3)	
H19	1.1826	1.0054	0.5897	0.060*	
C20	1.1768 (12)	1.0467 (7)	0.4554 (9)	0.058 (3)	
H20	1.2259	1.1055	0.4727	0.070*	
C21	1.1315 (12)	1.0226 (6)	0.3587 (8)	0.049 (3)	
H21	1.1498	1.0646	0.3097	0.059*	
C22	1.0589 (11)	0.9361 (5)	0.3345 (7)	0.037 (2)	
H22	1.0272	0.9187	0.2688	0.044*	
C23	1.0596 (12)	0.8356 (7)	0.5848 (6)	0.045 (2)	
H23A	1.1387	0.7822	0.5831	0.068*	
H23B	0.9325	0.8154	0.5782	0.068*	
H23C	1.0941	0.8677	0.6462	0.068*	
C24	1.1434 (10)	0.7751 (5)	0.0239 (6)	0.0267 (18)	
C25	1.0177 (10)	0.8323 (5)	−0.0341 (5)	0.0276 (18)	
C26	0.9738 (11)	0.8081 (7)	−0.1314 (6)	0.042 (2)	
H26	0.8901	0.8452	−0.1730	0.050*	
C27	1.0464 (13)	0.7330 (6)	−0.1693 (6)	0.045 (2)	
H27	1.0144	0.7192	−0.2360	0.054*	
C28	1.1654 (12)	0.6781 (6)	−0.1103 (7)	0.044 (2)	
H28	1.2144	0.6253	−0.1359	0.053*	
C29	1.2146 (10)	0.6990 (6)	−0.0141 (6)	0.0334 (17)	
H29	1.2980	0.6608	0.0263	0.040*	
C30	0.9353 (11)	0.9151 (6)	0.0057 (7)	0.040 (2)	
H30A	1.0317	0.9593	0.0289	0.060*	
H30B	0.8745	0.8965	0.0593	0.060*	
H30C	0.8462	0.9432	−0.0451	0.060*	

### Atomic displacement parameters (Å^2^)

**Table:**

	*U*^11^	*U*^22^	*U*^33^	*U*^12^	*U*^13^	*U*^23^
In1	0.0262 (3)	0.0223 (2)	0.0294 (3)	−0.0009 (2)	0.0116 (2)	−0.0015 (3)
In2	0.0220 (3)	0.0244 (3)	0.0273 (3)	−0.0018 (2)	0.0085 (2)	−0.0032 (2)
S1	0.0209 (9)	0.0376 (11)	0.0221 (10)	−0.0070 (10)	0.0047 (8)	−0.0036 (10)
S2	0.0205 (10)	0.0232 (9)	0.0225 (10)	−0.0030 (7)	0.0057 (8)	−0.0025 (8)
S3	0.0218 (10)	0.0283 (9)	0.0203 (10)	−0.0009 (8)	0.0062 (9)	−0.0015 (8)
S4	0.0204 (9)	0.0356 (10)	0.0244 (10)	−0.0058 (11)	0.0039 (8)	0.0023 (11)
C1	0.033 (5)	0.031 (5)	0.042 (5)	−0.004 (4)	0.003 (4)	−0.006 (4)
C2	0.027 (5)	0.021 (4)	0.053 (6)	0.006 (3)	0.005 (4)	−0.004 (4)
C3	0.016 (4)	0.026 (3)	0.029 (4)	0.007 (3)	0.006 (3)	0.000 (3)
C4	0.021 (4)	0.033 (4)	0.027 (4)	0.005 (3)	0.004 (3)	0.002 (3)
C5	0.044 (5)	0.033 (4)	0.027 (4)	0.005 (4)	0.008 (4)	0.005 (4)
C6	0.035 (5)	0.044 (4)	0.025 (5)	0.001 (4)	0.005 (4)	−0.002 (4)
C7	0.033 (5)	0.032 (4)	0.037 (5)	−0.008 (4)	0.008 (4)	−0.014 (4)
C8	0.027 (4)	0.032 (4)	0.032 (4)	0.001 (4)	0.010 (3)	−0.002 (4)
C9	0.059 (6)	0.039 (5)	0.022 (5)	−0.011 (4)	0.004 (4)	0.002 (4)
C10	0.010 (4)	0.024 (4)	0.025 (4)	0.002 (3)	0.005 (3)	−0.002 (3)
C11	0.017 (4)	0.031 (4)	0.030 (4)	0.006 (3)	0.009 (3)	−0.003 (3)
C12	0.027 (4)	0.029 (4)	0.036 (5)	0.003 (4)	0.002 (4)	−0.012 (4)
C13	0.032 (5)	0.021 (4)	0.052 (6)	−0.009 (4)	0.008 (4)	0.000 (4)
C14	0.029 (5)	0.026 (4)	0.040 (5)	0.000 (3)	0.016 (4)	0.001 (4)
C15	0.031 (5)	0.030 (4)	0.025 (4)	−0.004 (4)	0.009 (4)	0.001 (3)
C16	0.040 (5)	0.041 (5)	0.032 (5)	−0.001 (4)	0.010 (4)	0.000 (4)
C17	0.018 (4)	0.029 (4)	0.038 (5)	−0.002 (3)	0.007 (4)	−0.008 (4)
C18	0.013 (4)	0.046 (5)	0.054 (6)	0.008 (4)	0.007 (4)	−0.022 (5)
C19	0.026 (5)	0.066 (7)	0.058 (7)	−0.005 (5)	0.005 (5)	−0.037 (6)
C20	0.026 (5)	0.054 (6)	0.096 (10)	−0.011 (5)	0.015 (6)	−0.036 (7)
C21	0.036 (5)	0.035 (5)	0.081 (8)	−0.008 (4)	0.021 (5)	−0.012 (5)
C22	0.034 (5)	0.029 (4)	0.052 (6)	−0.007 (4)	0.023 (4)	−0.009 (4)
C23	0.037 (5)	0.071 (6)	0.027 (5)	0.012 (5)	0.003 (4)	−0.007 (4)
C24	0.023 (4)	0.035 (4)	0.024 (4)	−0.007 (3)	0.011 (3)	0.003 (3)
C25	0.024 (4)	0.033 (4)	0.025 (4)	−0.010 (3)	0.002 (3)	0.005 (3)
C26	0.039 (5)	0.049 (5)	0.034 (5)	−0.013 (5)	−0.006 (4)	0.015 (5)
C27	0.053 (6)	0.061 (6)	0.020 (5)	−0.028 (5)	0.004 (4)	−0.002 (4)
C28	0.048 (6)	0.044 (6)	0.043 (6)	−0.011 (5)	0.017 (5)	−0.017 (4)
C29	0.022 (4)	0.042 (4)	0.038 (5)	−0.003 (4)	0.009 (3)	0.000 (5)
C30	0.027 (5)	0.035 (4)	0.055 (6)	0.004 (4)	−0.002 (4)	0.015 (4)

### Geometric parameters (Å, º)

**Table:**

In1—C1	2.119 (9)	C12—C13	1.366 (12)
In1—S1	2.466 (2)	C12—H12	0.9500
In1—S2	2.531 (2)	C13—C14	1.379 (11)
In1—S3	2.678 (2)	C13—H13	0.9500
In1—S4^i^	3.0910 (19)	C14—C15	1.398 (10)
In2—C2	2.146 (8)	C14—H14	0.9500
In2—S4	2.460 (2)	C15—H15	0.9500
In2—S3	2.546 (2)	C16—H16A	0.9800
In2—S2	2.722 (2)	C16—H16B	0.9800
In2—S1^ii^	2.9201 (19)	C16—H16C	0.9800
S1—C3	1.776 (8)	C17—C22	1.386 (12)
S1—In2^i^	2.9201 (19)	C17—C18	1.386 (12)
S2—C10	1.794 (7)	C18—C19	1.415 (12)
S3—C17	1.802 (8)	C18—C23	1.491 (13)
S4—C24	1.792 (8)	C19—C20	1.339 (15)
C1—H1A	0.9800	C19—H19	0.9500
C1—H1B	0.9800	C20—C21	1.391 (14)
C1—H1C	0.9800	C20—H20	0.9500
C2—H2A	0.9800	C21—C22	1.395 (11)
C2—H2B	0.9800	C21—H21	0.9500
C2—H2C	0.9800	C22—H22	0.9500
C3—C8	1.386 (10)	C23—H23A	0.9800
C3—C4	1.404 (10)	C23—H23B	0.9800
C4—C5	1.398 (11)	C23—H23C	0.9800
C4—C9	1.504 (11)	C24—C29	1.379 (11)
C5—C6	1.361 (11)	C24—C25	1.409 (10)
C5—H5	0.9500	C25—C26	1.399 (11)
C6—C7	1.373 (11)	C25—C30	1.508 (11)
C6—H6	0.9500	C26—C27	1.371 (13)
C7—C8	1.393 (11)	C26—H26	0.9500
C7—H7	0.9500	C27—C28	1.368 (13)
C8—H8	0.9500	C27—H27	0.9500
C9—H9A	0.9800	C28—C29	1.376 (11)
C9—H9B	0.9800	C28—H28	0.9500
C9—H9C	0.9800	C29—H29	0.9500
C10—C15	1.374 (10)	C30—H30A	0.9800
C10—C11	1.409 (10)	C30—H30B	0.9800
C11—C12	1.393 (10)	C30—H30C	0.9800
C11—C16	1.500 (11)		
			
C1—In1—S1	127.3 (2)	C12—C11—C10	116.6 (7)
C1—In1—S2	113.1 (3)	C12—C11—C16	121.7 (8)
S1—In1—S2	114.66 (7)	C10—C11—C16	121.6 (7)
C1—In1—S3	105.7 (2)	C13—C12—C11	122.9 (8)
S1—In1—S3	96.94 (6)	C13—C12—H12	118.6
S2—In1—S3	88.28 (6)	C11—C12—H12	118.6
C1—In1—S4^i^	85.4 (2)	C12—C13—C14	119.8 (7)
S1—In1—S4^i^	73.86 (6)	C12—C13—H13	120.1
S2—In1—S4^i^	89.64 (6)	C14—C13—H13	120.1
S3—In1—S4^i^	168.67 (6)	C13—C14—C15	119.3 (8)
C2—In2—S4	124.1 (3)	C13—C14—H14	120.4
C2—In2—S3	118.2 (3)	C15—C14—H14	120.4
S4—In2—S3	115.00 (7)	C10—C15—C14	120.4 (8)
C2—In2—S2	102.4 (2)	C10—C15—H15	119.8
S4—In2—S2	95.87 (6)	C14—C15—H15	119.8
S3—In2—S2	87.02 (6)	C11—C16—H16A	109.5
C2—In2—S1^ii^	88.3 (2)	C11—C16—H16B	109.5
S4—In2—S1^ii^	77.21 (6)	H16A—C16—H16B	109.5
S3—In2—S1^ii^	88.45 (6)	C11—C16—H16C	109.5
S2—In2—S1^ii^	169.21 (6)	H16A—C16—H16C	109.5
C3—S1—In1	109.9 (3)	H16B—C16—H16C	109.5
C3—S1—In2^i^	125.0 (3)	C22—C17—C18	122.0 (8)
In1—S1—In2^i^	106.71 (7)	C22—C17—S3	120.2 (6)
C10—S2—In1	111.6 (2)	C18—C17—S3	117.8 (6)
C10—S2—In2	98.4 (2)	C17—C18—C19	116.1 (9)
In1—S2—In2	91.86 (6)	C17—C18—C23	124.3 (8)
C17—S3—In2	109.2 (3)	C19—C18—C23	119.6 (8)
C17—S3—In1	105.3 (3)	C20—C19—C18	123.0 (9)
In2—S3—In1	92.58 (7)	C20—C19—H19	118.5
C24—S4—In2	103.5 (2)	C18—C19—H19	118.5
In1—C1—H1A	109.5	C19—C20—C21	120.1 (9)
In1—C1—H1B	109.5	C19—C20—H20	120.0
H1A—C1—H1B	109.5	C21—C20—H20	120.0
In1—C1—H1C	109.5	C20—C21—C22	119.2 (10)
H1A—C1—H1C	109.5	C20—C21—H21	120.4
H1B—C1—H1C	109.5	C22—C21—H21	120.4
In2—C2—H2A	109.5	C17—C22—C21	119.5 (9)
In2—C2—H2B	109.5	C17—C22—H22	120.2
H2A—C2—H2B	109.5	C21—C22—H22	120.2
In2—C2—H2C	109.5	C18—C23—H23A	109.5
H2A—C2—H2C	109.5	C18—C23—H23B	109.5
H2B—C2—H2C	109.5	H23A—C23—H23B	109.5
C8—C3—C4	120.1 (7)	C18—C23—H23C	109.5
C8—C3—S1	122.8 (6)	H23A—C23—H23C	109.5
C4—C3—S1	117.0 (6)	H23B—C23—H23C	109.5
C5—C4—C3	117.4 (7)	C29—C24—C25	121.0 (7)
C5—C4—C9	120.9 (7)	C29—C24—S4	119.9 (6)
C3—C4—C9	121.6 (7)	C25—C24—S4	119.0 (6)
C6—C5—C4	122.2 (8)	C26—C25—C24	116.1 (8)
C6—C5—H5	118.9	C26—C25—C30	121.7 (8)
C4—C5—H5	118.9	C24—C25—C30	122.2 (7)
C5—C6—C7	120.2 (8)	C27—C26—C25	122.8 (8)
C5—C6—H6	119.9	C27—C26—H26	118.6
C7—C6—H6	119.9	C25—C26—H26	118.6
C6—C7—C8	119.5 (7)	C28—C27—C26	119.4 (8)
C6—C7—H7	120.3	C28—C27—H27	120.3
C8—C7—H7	120.3	C26—C27—H27	120.3
C3—C8—C7	120.4 (7)	C27—C28—C29	120.3 (9)
C3—C8—H8	119.8	C27—C28—H28	119.8
C7—C8—H8	119.8	C29—C28—H28	119.8
C4—C9—H9A	109.5	C28—C29—C24	120.3 (8)
C4—C9—H9B	109.5	C28—C29—H29	119.8
H9A—C9—H9B	109.5	C24—C29—H29	119.8
C4—C9—H9C	109.5	C25—C30—H30A	109.5
H9A—C9—H9C	109.5	C25—C30—H30B	109.5
H9B—C9—H9C	109.5	H30A—C30—H30B	109.5
C15—C10—C11	121.1 (7)	C25—C30—H30C	109.5
C15—C10—S2	121.1 (6)	H30A—C30—H30C	109.5
C11—C10—S2	117.7 (6)	H30B—C30—H30C	109.5

Symmetry codes: (i) *x*−1, *y*, *z*; (ii) *x*+1, *y*, *z*.
